# Robot‐assisted radical nephrectomy and inferior vena cava tumor thrombectomy using the novel surgical robot platform, hinotori: Initial experience with two cases

**DOI:** 10.1002/iju5.12673

**Published:** 2023-11-21

**Authors:** Daisuke Motoyama, Yuto Matsushita, Hiromitsu Watanabe, Keita Tamura, Atsushi Otsuka, Masato Fujisawa, Hideaki Miyake

**Affiliations:** ^1^ Department of Urology Hamamatsu University School of Medicine Hamamatsu Japan; ^2^ Department of Developed Studies for Advanced Robotic Surgery Hamamatsu University School of Medicine Hamamatsu Japan; ^3^ Division of Urology Kobe University Graduate School of Medicine Kobe Japan

**Keywords:** hinotori, inferior vena cava tumor thrombectomy, renal cell carcinoma, robot‐assisted radical nephrectomy

## Abstract

**Introduction:**

A newly developed surgical robot system, hinotori, with various unique advantages has been in clinical use in Japan; however, there have not been any studies of robot‐assisted radical nephrectomy and inferior vena cava tumor thrombectomy using hinotori.

**Case presentation:**

We describe two male patients aged 67 and 76 years old with right renal cell carcinoma and a level II and I inferior vena cava tumor thrombus, respectively, undergoing robot‐assisted radical nephrectomy and inferior vena cava tumor thrombectomy using hinotori. Both operations were successfully completed with a purely robotic procedure without any major perioperative complications, resulting in the following findings: time using robotic system, 158 and 156 min; total operative time, 228 and 214 min; estimated blood loss, 535 and 200 mL, respectively.

**Conclusion:**

Based on our first experience, robot‐assisted radical nephrectomy and inferior vena cava tumor thrombectomy using hinotori may be an effective treatment for renal cell carcinoma with inferior vena cava tumor thrombus ≤level II.

**Abbreviations & Acronyms**
IVC: inferior vena cava
RARN/IVCTT: robot‐assisted radical nephrectomy and inferior vena cava tumor thrombectomy
RCC: renal cell carcinoma
RN: radical nephrectomy
WHO/ISUP: World Health Organization/International Society of Urological Pathology

**Keynote message**

This is the first case report presenting two patients receiving RARN/IVCTT using newly developed hinotori surgical system, and our experience suggests that purely robotic surgery using hinotori may be a useful treatment for RCC with IVC tumor thrombus ≤level II.

## Introduction

Although open RN and IVCTT remain the standard approach for patients with RCC with IVC tumor thrombus,^1^ recent progress in robotic technologies has led to the application of robotic surgery to these patients.^2^ Since the first study in 2011,^3^ promising outcomes of RARN/IVCTT have been reported in several studies at high‐volume centers.^2^, ^3^, ^4^, ^5^, ^6^, ^7^

The da Vinci surgical system (Intuitive Surgical Inc., Sunnyvale, CA, USA) has monopolized the surgical robot market for the past 2 decades. On the other hand, numerous alternative robotic systems are being actively developed, and some are in clinical use.^8^, ^9^, ^10^, ^11^, ^12^, ^13^, ^14^ Among them, hinotori, launched in 2019 by the Medicaloid Corporation (Kobe, Japan), has unique advantages that are different from existing platforms.^12^

To our knowledge, there have been no studies of RARN/IVCTT using hinotori. The objective of the present report was to summarize the initial experience of RARN/IVCTT using hinotori for two patients with right RCC and IVC tumor thrombus corresponding to levels I and II, based on the Mayo clinic classification.^1^

## Case presentation

### Case 1

**Fig. 1:**
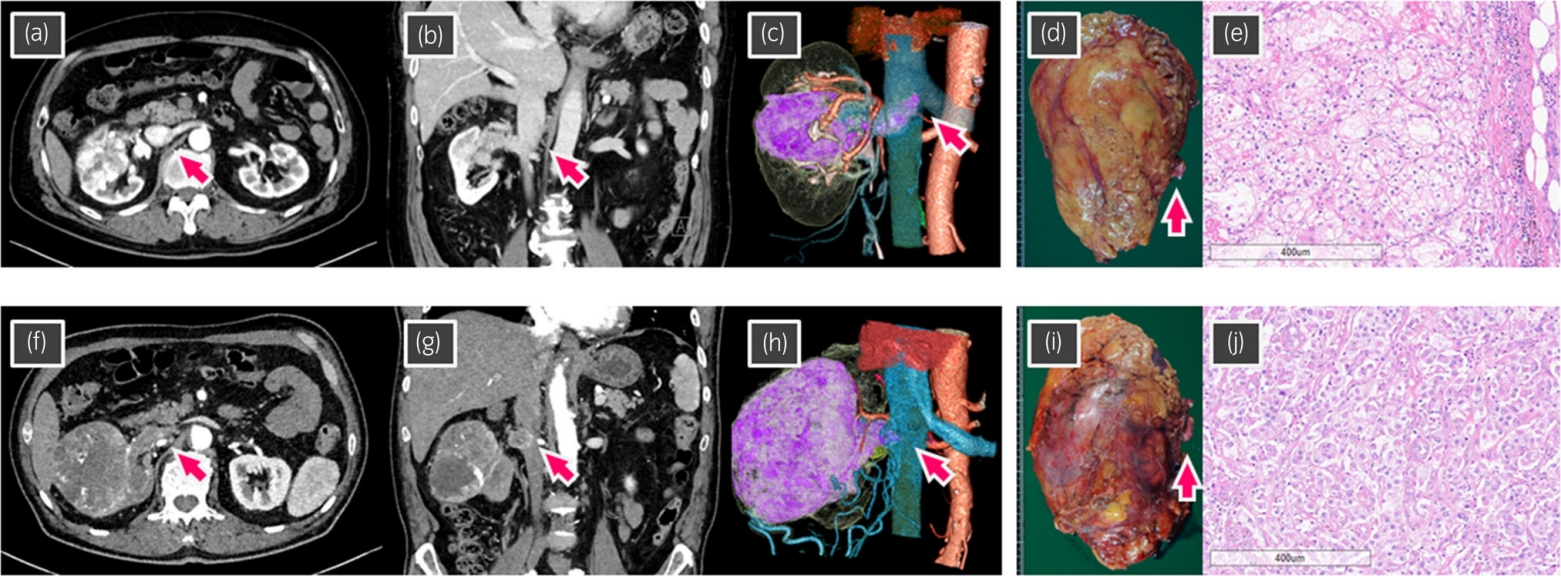
(a–e) Case 1 showing right RCC with a level II IVC tumor thrombus (arrow). (a) Axial section of computed tomography (CT). (b) Coronal section of CT. (c) Three‐dimensional reconstructed image from CT. (d), Macroscopic findings of the excised right kidney and IVC tumor thrombus (arrow). Excised weight was 595 g. (e) Microscopic findings of hematoxylin and eosin staining showing clear cell RCC, pT3b, WHO/ISUP grade 3. (f–j) Case 2 showing right RCC with level I IVC tumor thrombus (arrow). (f) Axial section of CT. (g) Coronal section of CT. (h) Three‐dimensional reconstructed image from CT. (i), Macroscopic findings of the excised right kidney and IVC tumor thrombus (arrow). Excised weight was 635 g. (j), Microscopic findings of hematoxylin and eosin staining showing clear cell RCC, pT3b, WHO/ISUP grade 4.

A 67‐year‐old male patient presented with a right renal mass and was referred to our institution. An enhanced right renal tumor with a level II IVC tumor thrombus (22 mm high from the right renal vein) without evidence of distant metastasis was observed on computed tomography (Table 1; Fig. 1). We administered 20 mg of cabozantinib daily as presurgical medication. After 4 weeks, the size of the right renal tumor decreased from 78 to 71 mm in diameter, while the height of the IVC tumor thrombus was not changed. Considering the slight reduction of the tumor and favorable condition of the patient, RARN/IVCTT using hinotori was planned.

**Table 1:** Perioperative characteristics of two patients who underwent robot‐assisted RN and IVC thrombectomy using hinotori surgical robot system

Variables	Case 1	Case 2
Age (years)	67	79
Sex	Male	Male
Body mass index (kg/m^2^)	27.1	22.1
ECOG‐PS	0	0
ASA score	II	I
History of abdominal surgery	No	No
Tumor side	Right	Right
Tumor size (mm)	78	106
TNM classification	cT3bN0M0	cT3bN0M0
Distance of thrombus above the renal vein (mm)	22	4
Level of IVC thrombus	Level II	Level I
Presurgical medication	Cabozantinib 20 mg x4weeks	No
Surgical approach	Transperitoneal	Transperitoneal
Operative time (min)	228	214
Time using robotic system (min)	158	156
Estimated blood loss (mL)	535	200
Blood translation	No	No
Open conversion	No	No
Postoperative major complication (Clavien‐Dindo ≥3)	No	No
Length of postoperative hospital stay (days)	8	10
Resected weight (g)	595	635
Histological subtype	Clear cell RCC	Clear cell RCC
pT stage	pT3b	pT3b
WHO/ISUP grading	Grade 3	Grade 4

ASA, American Society of Anesthesiologists; ECOG‐PS, Eastern Cooperative Oncology Group Performance Status.

Surgical procedure including the operative position and trocar placement were the same as those in our previous report using da Vinci Xi.^15^, ^16^ Time using the robotic system, total operating time, and estimated blood loss were 158, 228 min, and 535 mL, respectively. No major complications occurred, and blood transfusion during and after RARN/IVCTT was not required. On the eighth day after surgery, the patient was discharged from the hospital. The following findings were revealed by pathological assessment: clear cell RCC, pT3b, and WHO/ISUP grade 3 (Table 1).

### Case 2

**Fig. 2:**
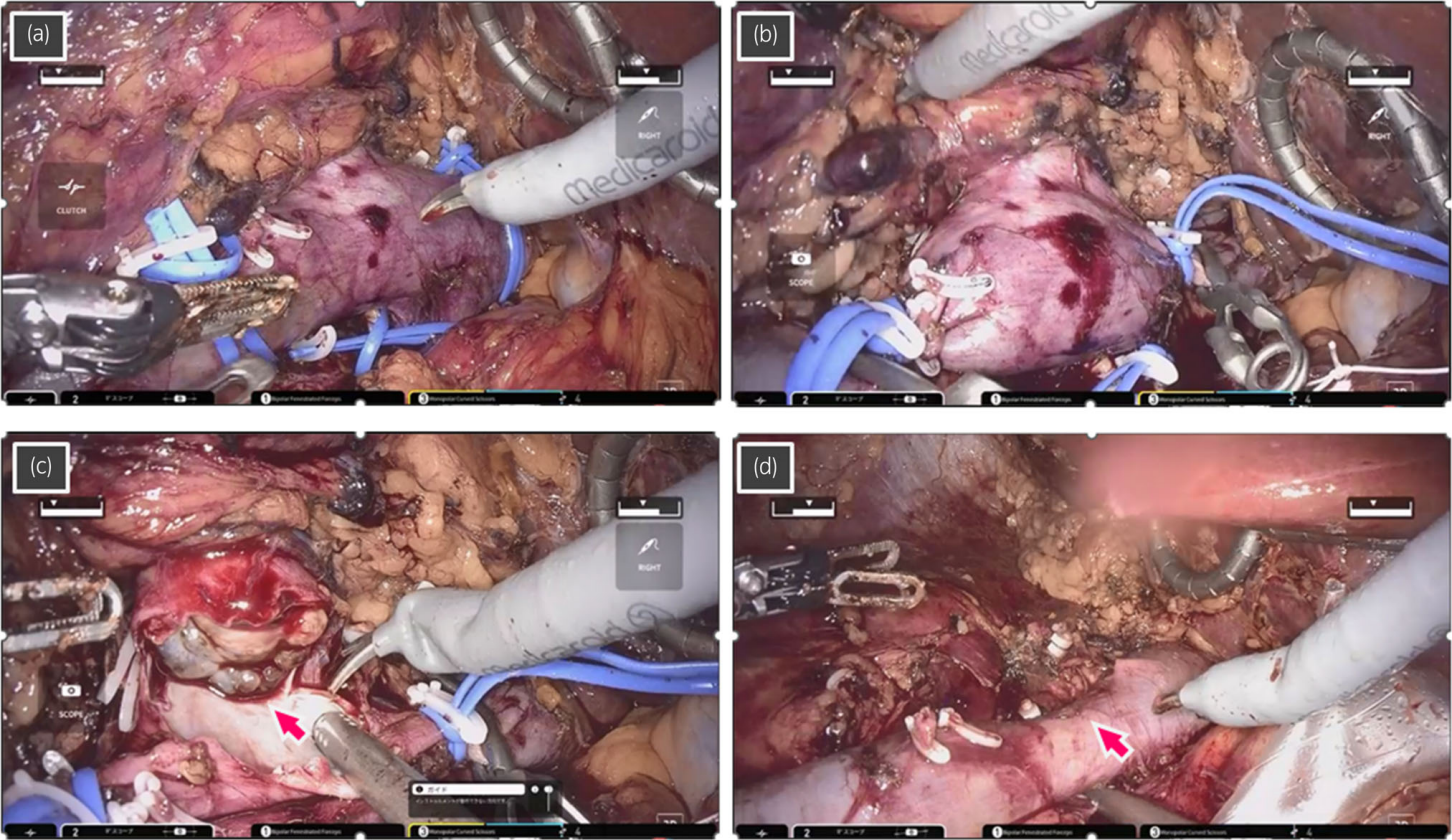
Intraoperative images during robot‐assisted RN and IVC tumor thrombectomy for case 2 who had level I IVC tumor thrombus. (a) The left renal vein, caudal IVC, and cephalic IVC are secured by twice‐wrapped vessel loops, and (b) sequentially clamped with the vessel loops by clipping in addition to the use of bulldogs. (c) Tumor thrombus (arrow) is removed from the IVC, and the wall of IVC is cut. (d) The IVC is reconstructed by continuous suture with a 4‐0 polypropylene (arrow), following removal of the tumor thrombus.

A 67‐year‐old male patient with right renal tumor (106 mm in diameter) and a level I IVC tumor thrombus (4 mm high from the right renal vein) was referred to our institution. Clinical stage was diagnosed as cT3bN0M0 (Table 1, Fig. 1). RARN/IVCTT using hinotori was performed without presurgical therapy using surgical procedures similar to those in case 1 (Fig. 2).

Time using the robotic system, total operating time, and estimated blood loss were 156, 214 min, and 200 mL, respectively. The surgery was conducted with a purely robotic approach without open conversion, major complications, or blood transfusion, and duration of hospital stay after surgery was 10 days. The following findings were revealed by pathological assessment: clear cell RCC, pT3b, and WHO/ISUP grade 4.

## Discussion

Since the initial study,^3^ robot‐assisted surgery for RCC with IVC tumor thrombus have increased as a minimally invasive procedure alternative to the conventional open approach, since the outcomes of RARN/IVCTT have been reported to be significantly less intraoperative blood loss and complications compared to open approaches.^2^, ^3^, ^4^, ^5^, ^6^, ^7^ Thus, the robotic surgery has been extended to RCC patients with a higher level of IVC tumor thrombus by a small number of limited institutions.^17^, ^18^ However, it remains unclear whether novel robotic platforms, including hinotori, can be safely used for RARN/IVCTT. To the best of our knowledge, this may be the first report of purely robotic RARN/IVCTT procedures being successfully completed using hinotori.

Hinotori has several unique features that make it different from the existing da Vinci system, such as a flexibly positionable three‐dimensional viewer in the surgeon cockpit, calibration of the position of trocars by software without docking of an arm with a port, and flexible movement of compactly designed robotic arms with 8 axes of motion.^12^ In Japan, several fields of surgery, including urology, gynecology, and gastroenterology, have introduced the hinotori in daily clinical practice. In the present report, considering the promising features of its equipment and clinical performance, we performed RARN/IVCTT using hinotori for two surgeries.

The RARN/IVCTT surgeries using hinotori were completed without any major perioperative complications according to the procedures same as those in cases using da Vinci since these two surgical robot systems are characterized by the fundamentally common structural design, consisting of an operation unit, surgeon cockpit, and monitor cart. The time using the robotic system was approximately 150 min for both surgeries, which is shorter than previous studies that used da Vinci.^2^, ^3^, ^4^, ^5^, ^6^, ^7^ These findings are similar to the console times reported in our previous study of two cases with IVC tumor thrombus receiving RARN/IVCTT with da Vinci (167 and 233 min, respectively).^16^ In addition, other perioperative outcomes with hinotori, including estimated blood loss and length of postoperative hospital stay, were comparable to those with da Vinci in our experienced cases. Collectively, these findings suggest that for cases with IVC tumor thrombus ≤infrahepatic level II, RARN/IVCTT using hinotori can achieve similar perioperative outcomes to da Vinci.

This study had several limitations. Firstly, it was required to consider whether procedures omitted in these cases, such as covering of the removed thrombus by using a specimen bag and irrigation of the caval lumen, should be performed. Secondly, length of hospital stay in Japan tends to be longer compared to Western countries regardless of surgical procedure. Thirdly, cabozantinib was used as a presurgical medication in case 1; however, since the efficacy of presurgical medication remains controversial in the field of RCC, this indication should be carefully determined. Finally, further studies are needed for the application of the hinotori for patients with an IVC tumor thrombus ≥level III or all levels of IVC tumor thrombus with left‐sided renal tumor.

In conclusion, this is the initial report presenting successful completion of RARN/IVCTT for two cases with level I and II IVC tumor thrombus using hinotori. Purely robotic surgery using hinotori may be an effective treatment for such patients.

## Author contributions

Daisuke Motoyama: Data curation; formal analysis; investigation; methodology; project administration; resources; validation; visualization; writing – original draft. Yuto Matsushita: Investigation. Hiromitsu Watanabe: Investigation. Keita Tamura: Investigation. Atsushi Otsuka: Investigation. Masato Fujisawa: Supervision. Hideaki Miyake: Conceptualization; investigation; writing – review and editing.

## Conflict of interest

The authors have no conflict of interest.

## Approval of the research protocol by an Institutional Reviewer Board

21‐091.

## Informed consent

Not applicable.

## Registry and the Registration No. of the study/trial

Not applicable.
